# Symmetric Talbot-Lau neutron grating interferometry and incoherent scattering correction for quantitative dark-field imaging

**DOI:** 10.1038/s41598-019-55420-3

**Published:** 2019-12-12

**Authors:** Youngju Kim, Jacopo Valsecchi, Jongyul Kim, Seung Wook Lee, Markus Strobl

**Affiliations:** 1 School of Mechanical Engineering, Pusan National University, Busan, Republic of Korea; 2 Laboratory for Neutron Scattering and Imaging, Paul Scherrer Institut, Villigen, Switzerland; 3 University of Geneva, Geneva, Switzerland; 4 Neutron Science Center, Korea Atomic Energy Research Institute, Daejeon, Republic of Korea

**Keywords:** Engineering, Materials science, Optics and photonics

## Abstract

We introduce the application of a symmetric Talbot-Lau neutron grating interferometer which provides a significantly extended autocorrelation length range essential for quantitative dark-field contrast imaging. The highly efficient set-up overcomes the limitation of the conventional Talbot-Lau technique to a severely limited micrometer range as well as the limitation of the other advanced dark-field imaging techniques in the nanometer regime. The novel set-up enables efficient and continuous dark-field contrast imaging providing quantitative small-angle neutron scattering information for structures in a regime from some tens of nanometers to several tens of micrometers. The quantitative analysis enabled in and by such an extended range is demonstrated through application to reference sample systems of the diluted polystyrene particle in aqueous solutions. Here we additionally demonstrate and successfully discuss the correction for incoherent scattering. This correction results to be necessary to achieve meaningful quantitative structural results. Furthermore, we present the measurements, data modelling and analysis of the two distinct kinds of cohesive powders enabled by the novel approach, revealing the significant structural differences of their fractal nature.

## Introduction

Dark-field imaging in neutron grating interferometry can be considered a novel analytical tool in material science providing spatially resolved local structural information of materials based on small-angle neutron scattering (SANS)^1,2^. The peculiarity of neutron dark-field imaging in material science is thus that it combines the regimes of neutron imaging and SANS. Neutron imaging provides macroscopic spatial information, i.e., resolving individual structural variations and inhomogeneities in a size range down to at least some tens of micrometers^3,4^, while SANS provides statistical structural information of materials from micrometers down to nanometers. Neutron dark-field imaging spans a combined size range from centimeters down to the nanometer range and from individual real space resolution in imaging to statistical Fourier space characterization in scattering.

Initially dark-field contrast has been utilized qualitatively, meaning to observe structural inhomogeneities. Already that has enabled a number of distinguished studies visualizing e.g. structural flaws in engineering materials^5–9^ and in particular magnetic domain structures in the volume of bulk materials^10–17^, where they are not assessable by any other means. However, based on the small-angle scattering theory a description of dark-field contrast became available^18^, which in analogy to spin-echo small-angle scattering, enables quantitative assessment of the scattering structures^19–24^. It can be shown that thus structures beyond the direct spatial resolution capabilities can be characterized locally, i.e. with image resolution. Like in small-angle scattering the main constituents to describe the scattering from a microscopic structure are the total small-angle scattering cross-section containing the volume fractions, scattering contrast of the contained material phases and the wavelength utilized. With respect to the structural morphology the structure has to be described by a one-dimensional projected real space correlation function *G*^18^. Data analysis in general involves fitting of model correlation functions to the measured projected real space correlation function, scaled by the scattering probability of the cross-section.

The availability of a quantitative theory enabling structural analysis unfolded the full potential of dark-field imaging for material science and structural characterization over a wide and unprecedented range of length scales with x-rays^25^ as well as with neutrons^26,27^. Examples of the latter include a study on the quasi-crystallization of micro-spheres through sedimentation^28^ and the spatially resolved investigation of the break-down of fractal structures in a cohesive powder under pressure^29^.

However, applications remain limited due to the limited range of autocorrelation lengths that can be probed with the standard neutron Talbot-Lau set-up^30–33^. Despite attempts to extend the range by increasing the Talbot order and hence the distance between the phase and analyzer grating the range hardly exceeds a few micrometers currently. Extending the range through variation of the wavelength is limited by the severe loss of visibility when moving away from the design wavelength of this monochromatic technique. Grating-based far-field interferometry^9,34^ and spin-echo modulated dark-field imaging^35^ enable to access different length scales in the nanometer regime, but these face different problems in extending to the micrometer scale in terms of sample-to-detector distances requiring extreme sample-to-detector distances impacting either spatial resolution or flux conditions severely.

In this paper, we demonstrate how a symmetric Talbot-Lau neutron grating interferometry can significantly extend the probed autocorrelation length range, enabling a significant improvement of the resolution range in small-angle scattering from some ten nanometers to some ten micrometers, i.e. three orders of magnitude. This is a precondition to enable meaningful structural models to be fitted with minimum a priori knowledge of the investigated systems. The symmetric set-up features large grating periods of some ten micrometer and long inter-grating distances^36–38^, demonstrating the possibility of approaching autocorrelation length of nanometer range. We demonstrate the superiority of our novel set-up through the application of quantitative dark-field imaging to well-known reference samples of the diluted polystyrene particle solutions. In addition, we investigate the need and introduce a correction for incoherent scattering from the aqueous phase which is required, in particular at short autocorrelation lengths for correct analysis of the structural features. Furthermore, we investigate two kinds of cohesive powders and demonstrate the significant structural differences of their fractal nature.

## Symmetric Talbot-Lau neutron Grating Interferometer

**Figure 1:**
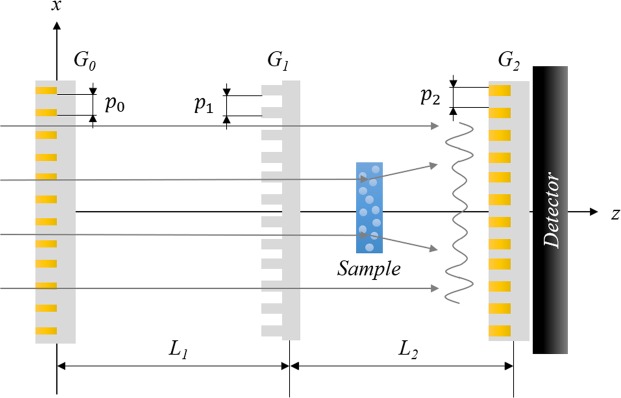
The schematics of symmetric Talbot-Lau neutron grating interferometer consists of a source grating (G_0_), a phase grating (G_1_), and an analyzer grating (G_2_). The inter-grating distance L_1_ and L_2_ are equal, and the set-up uses first fractional Talbot order. The sample is placed between G_1_ and G_2_ and moves along the z-axis for the variation of autocorrelation length of dark-field SANS imaging.

The principle of a Talbot-Lau neutron grating interferometer is shown in Fig. 1. The Talbot-Lau neutron interferometer consists of a source grating (G_0_), a phase grating (G_1_), and an analyzer grating (G_2_). The G_0_ is an absorption grating of gadolinium (Gd) or gadolinium oxysulfide (Gadox) and it is positioned close to the neutron source to fulfill the coherence requirements for the neutron beam in the interferometer. It generates spatial coherence by generating a number of independent beams, each with sufficient coherence exceeding the period of the phase grating. G_1_ is a silicon phase grating which generates an interference pattern for each individual beam from G_0_, which are due to the set-up geometry constructively superimposed at the analyzer grating position to form the Talbot pattern. The G_2_ is another absorption grating which is positioned at the first Talbot distance, in the symmetric set-up matching at the same time the G_0_-to-G_1_ distance. It is placed just in front of the detector and enables to resolve the Talbot pattern by matching its period. Thus, the Talbot pattern is resolved in a stepping procedure in every pixel of the detector, even when the detector resolution is not sufficient to directly resolve the pattern. A slight misalignment of G_2_ with respect to the modulation creates a moiré pattern which is resolved directly by the detector and supports alignment. However, the moiré pattern can in principle also be used to analyze the effects on the Talbot pattern by differential phase and scattering effects.

The geometry of Talbot-Lau neutron interferometers can be classified into three types, i.e. conventional, symmetric, and inverse geometry by the position of G_1_ between G_0_ and G_2_ and the corresponding periods of the gratings^39^. In the conventional type G_1_ is placed significantly closer to G_2_ than to G_0_, and it generally features several hundreds of micrometers for G_0_ period, and just several micrometers for periods of G_1_ and G_2_. In the symmetric type geometry G_1_ is placed at the middle-positioned between G_0_ and G_2_, and all grating periods are equal, in our first attempt described here some tens of micrometers. The inverse type is reversed with respect to the conventional case for the inter-grating distances and the periods of gratings. To our best knowledge the latter has never been attempted with neutrons so far.

It can be shown that the symmetric type has significant advantages compared to the conventional and currently standard type of neutron Talbot-Lau interferometer. Firstly, the symmetric type provides a great distance between G_1_ and G_2_ where the sample is best placed for quantitative dark-field contrast imaging and where conventionally the space is limited to few centimeters with a steep change of autocorrelation length probed. In addition, due to the relatively large grating periods, the symmetric set-up overcomes difficulties in grating fabrication. However, these purely geometric advantages have benefits in particular for the quantitative dark-field imaging with SANS analysis as will be outlined below.

## Experiments

The symmetric Talbot-Lau neutron grating interferometer for this research was applied at the cold neutron imaging beamline ICON^40^ of Paul Scherrer Institute. The details of the interferometer are presented in Table 1. G_0_ has the one-dimensional line structures of Gadox, G_1_ has two-dimensional checkerboard structures of silicon, and G_2_ has two-dimensional mesh grid structures of Gadox. The one-dimensional spatial coherence was defined by the line structure of G_0_, thus the interferometer has one-dimensional directional sensitivity^37^. It was operated with a monochromatic neutron beam of 4.4 Å using the available velocity selector with a wavelength resolution of about 12%. The detector was an Andor iKon-L CCD camera with commercial lens system. The scintillator was a 200 μm thick LiF:ZnS screen. The phase stepping method using the mechanical motor of G_0_ was conducted with 13 steps, and four exposures per step were merged by a median filter procedure. The exposure time was 100 seconds for each raw image.

**Table 1:** The details of symmetric Talbot-Lau neutron grating interferometer.

Wavelength (Å)	λ	4.4
Inter-grating distance (mm)	G_0_-G_1_ (L_1_)	1420
	G_1_-G_2_ (L_2_)	1420
Period of gratings (μm)	p (p_0_ = p_1_ = p_2_)	50
Height of gratings (μm)	h_0_	100 (Gadox)
	h_1_	34.39 (Silicon)
	h_2_	20 (Gadox)
Duty cycle of gratings	d_0_	0.75 (Gadox)
	d_1_	0.5 (Silicon)
	d_2_	0.5 (Gadox)

### Samples

Two different kinds of sample systems have been probed in our experiments. As standard reference samples we measured several spherical monodisperse polystyrene particles diluted in water were used^26,27^. In addition, fractal powders with structural correlations over a wide range of length scales were studied, one of which is well characterized by SANS^29^.

The polystyrene particles are spheres in size of 0.15 μm, 0.31 μm, 0.6 μm, and 1 μm (The products are manufactured by Magsphere). The particles were diluted with the aqueous solution mixed with H_2_O and D_2_O (59 vol. %:41 vol. %) to achieve the homogeneous suspension of particles and the proper contrast, and each volume fraction of particles is 6 vol. %. The solutions were contained and sealed in the standard quartz cuvettes of 5 mm thickness. The difference of neutron scattering length density between polystyrene particles and solution at neutron wavelength of 4.4 Å is Δ*ρ*_0_ = 87.8 μm^−2^ for the polystyrene density of 1.05 g/cm^3^, the H_2_O density of 1.0 g/cm^3^, and the D_2_O density of 1.11 g/cm^3^.

The cohesive powders are Sipernat-310 and Sipernat-350 manufactured by EVONIK Industries AG. The powders consist of silica featuring particles in size of 8.5 μm and 4 μm respectively, and form a sponge-like agglomerate structure with numerous air pores of different sizes. The former is a high surface area silica with a wide pore size distribution and the latter is a macro-porous silica with low surface area. In the experiments the cohesive powders were contained and sealed in the quartz cuvettes of 5 mm thickness at air pressure. The difference of the neutron scattering length density between cohesive powders and air pores at a neutron wavelength of 4.4 Å is Δ*ρ*_0_ = 418.6 μm^−2^ for the silica density of 2.65 g/cm^3^.

### Data analysis

The dark-field SANS imaging is an imaging method measuring the quantitative structural information of materials based on SANS by resolving the change of dark-field contrast with respect to the autocorrelation lengths probed. The dark-field contrast is defined by the ratio of visibility of the modulation induced by the Talbot-Lau interferometer with the sample (*V*_*s*_) to the one without sample (*V*_*r*_). The resulting dark-field imaging (DFI) signal is expressed as a function of autocorrelation length by^18^:

1 $$DFI(\xi )={V}_{s}/{V}_{r}={e}^{{\varSigma }_{s}t(G(\xi )-1)},$$

where Σ_*s*_ is the total small-angle neutron scattering cross-section, *t* is the sample thickness, *ξ* is autocorrelation length, and *G*(*ξ*) is the one-dimensional projected real space correlation function describing the scattering structure. The total neutron scattering cross-section and projected real space correlation function are determined by the scattering structure and the materials. As this measurement technique is equivalent for every pixel of an image with the not-spatially resolved SANS technique, Spin-echo small-angle neutron scattering (SESANS), the analysis can build upon the principles and models for projected real space correlation functions *G* developed for SESANS. The autocorrelation length that is probed in a specific DFI measurement is a setup parameter of the grating interferometer and it is expressed by^18^:

2 $$\xi =\lambda {d}_{s}/{p}_{2},$$

where *λ* is the wavelength, *d*_*s*_ is the sample-to-G_2_ distance, and *p*_2_ is the period of G_2_. Correspondingly the measurement at a specific autocorrelation length probes the relative frequency of this autocorrelation length found in the scattering structure. The minimum and maximum autocorrelation lengths that can be probed by a set-up are the limits of the size range that can be measured through the DFI signal.

In the symmetric design, the range of autocorrelation length can be extended to not only towards smaller structures through the large period *p*_2_ but also by the significantly extended distance range possible between sample and G_2_, *d*_*s*_. Therefore, the symmetric Talbot-Lau neutron grating interferometer allows investigate through DFI structures from tens of nanometers to about 10 micrometers and beyond this through real space resolution up to centimeters.

The dark-field SANS imaging conducted scanned the autocorrelation length range probed by variation of the sample-to-G_2_ distance. The sample was moved stepwise from 13 mm to 689 mm distance from G_2_, resulting in an autocorrelation length range of 110 nm to 6.06 μm utilizing a modulation and hence G_2_ period of 50 μm and a wavelength of 4.4 Å. The diluted polystyrene particle solutions and cohesive powders were used for the experiments on the extended autocorrelation length range of the symmetric Talbot-Lau neutron grating interferometer.

### Diluted polystyrene particle solution (Isolated sphere model)

The monodisperse structure of the spherical particles diluted in water with low concentration can be described by an isolated sphere model, and the projected real space correlation function can be expressed by^22^:

3 $$G(\xi )={e}^{-\frac{9}{8}{(\frac{\xi }{r})}^{2}},$$

where *r* is the radius of sphere.

In this case, the total neutron scattering cross-section can be expressed by^27^:

4 $${\Sigma }_{s}=\frac{3}{2}{\lambda }^{2}\Delta {\rho }_{0}^{2}{\varphi }_{V}r,$$

where *φ*_*V*_ is the volume fraction (i.e. the concentration of particles).

### Cohesive powder (random two-phase media model)

A structure characterized by a pore distribution over a wide range of length scales such as the cohesive powder materials addressed here can be considered as a random two-phase media. In this case the correlation function in Eq. (1) may be expressed by^22^:

5 $$G(\xi )=\frac{2}{\varGamma (H+1/2)}{(\frac{\xi }{a})}^{H+1/2}{K}_{H+1/2}(\frac{\xi }{a}),$$

where Γ is the Gamma function, *H* is the Hurst exponent, *a* is the measure of size of heterogeneities, and *K*_*H*_ is the modified Bessel function of second kind. The Hurst exponent 0 ≤ *H* ≤ 1 represents the space-filling capacity (i.e. the dimensionality of fractal structure) of two-phase structure, so when *H*>1/2 the structure (i.e. the distribution of heterogeneities) has long-range correlated and smooth features, and when *H* < 1/2 the structure has short-range correlated and rough features. For the limits of Hurst exponent, when *H* = 0 represents a full space-filling and when *H* = 1 represents a smooth Euclidean distribution.

The total neutron scattering cross-section for random two-phase media is expressed by^21^:

6 $${\Sigma }_{s}={\lambda }^{2}\Delta {\rho }_{0}^{2}\phi (1-\phi ){\rm{\zeta }},$$

where Δ*ρ*_0_ is the neutron scattering length density contrast, *ϕ* is the packing fraction of two-phase media, and *ζ* is the corresponding characteristic autocorrelation length of the structure. Here, the characteristic autocorrelation length of the structure is expressed by^21^:

7 $$\zeta =\frac{2\sqrt{\pi }a\varGamma (H+1/2)}{\varGamma (H)}.$$

While Eq. (5) is a general case of a projected real space correlation function for a random two-phase media, it can be simplified e.g. when the structure is considered a perfectly random distribution of a solid material (here, *H* = 1/2). Then the projected real space correlation function can be expressed by^22^:

8 $$G(\xi )=(\frac{\xi }{a}){K}_{1}(\frac{\xi }{a}).$$

Another simplified case in particular for a fractal structure can be written as^22^:

9 $$G(\xi )={e}^{-{(\frac{\xi }{a})}^{\alpha }},$$

where 0 < *α* < 2 parametrizes the structure of the phase boundary. The parameter is related to the dimensional distribution, where 0 < *α* < 1 represents the structure of an open and branched distribution, while 1 < *α* < 2 represents a structure with a compact distribution. The limit of *α* = 1 represents a two-dimensional distribution corresponding to *H* = 0.

## Experimental Results and Discussions

**Figure 2:**
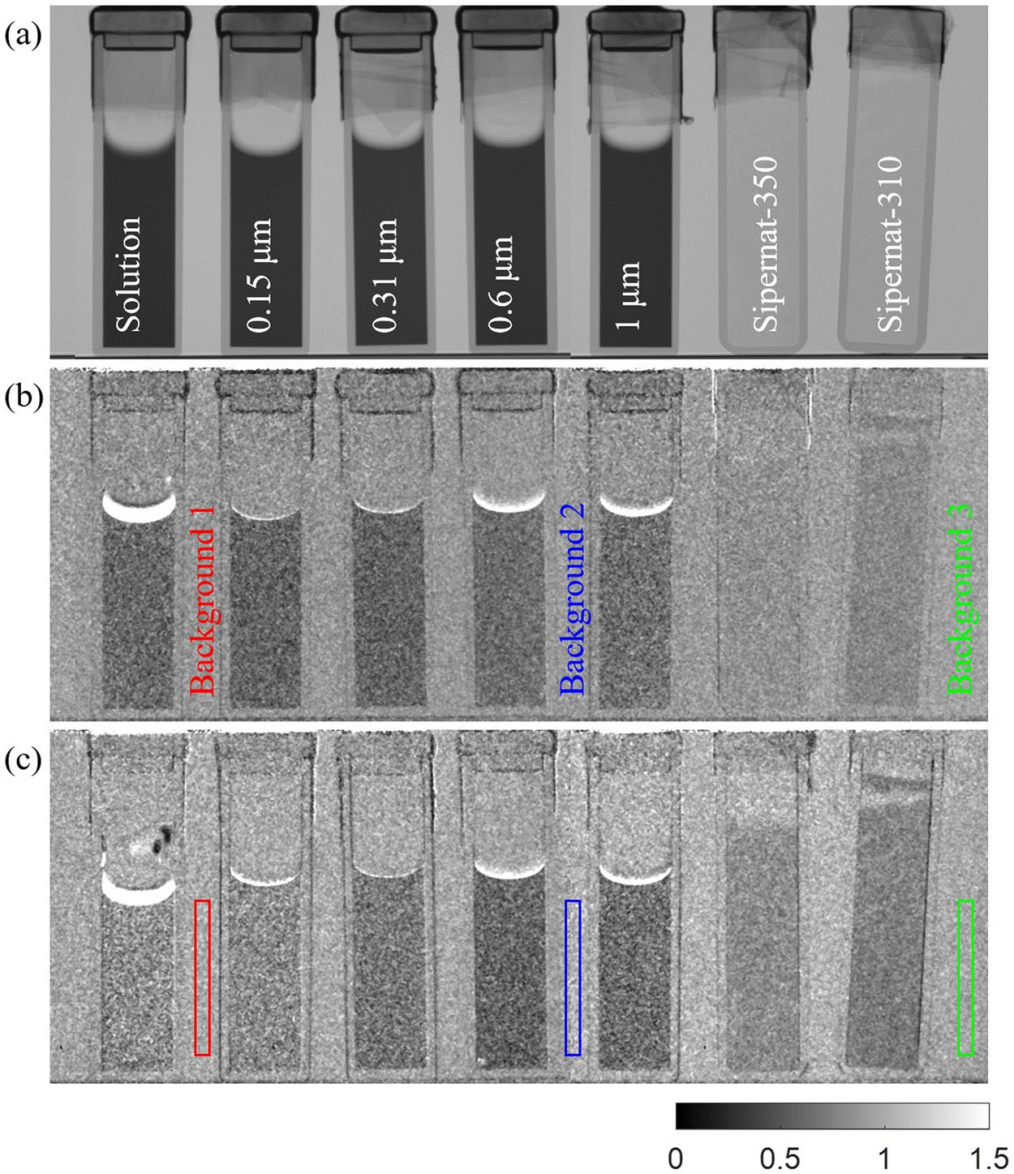
Result images of aqueous solution, diluted polystyrene particle solutions and cohesive powders of Sipernat-310 and Sipernnat-350. (**a**) Attenuation contrast image and (**b**) Dark-field contrast image are for the autocorrelation length of 110 nm. The dark-field contrasts in according sphere sizes are unresolvable at autocorrelation length of 110 nm due to incoherent neutron scattering of aqueous solution. (**c**) At longer autocorrelation length of 350 nm, the dark-field contrast image shows the different contrast for powder types and sphere sizes contrary to the attenuation contrast image.

The attenuation contrast and dark-field contrast images of pure aqueous solution, the diluted polystyrene particle solutions and the cohesive powders are shown in Fig. 2. The attenuation contrast (Fig. 2(a)) of the samples of the diluted polystyrene particles displays the same attenuation contrast for all the cuvettes, which is the same as for the pure aqueous solution in Fig. 2(a). The neutron beam is predominantly attenuated by the aqueous solution, and the attenuation contrast is only dependent to the thickness of the aqueous solution.

The dark-field contrast of the diluted polystyrene particle solutions and pure aqueous solution appear to have similar contrast in Fig. 2(b) measured at a short autocorrelation length parameter and hence with the sample close to G_2_ and the detector. The expected difference in SANS based dark-field contrast according to the different sphere sizes is not found. This is due to the dominance of the detected incoherent neutron scattering from H_2_O in the aqueous solution. At the probed autocorrelation length of 110 nm the sample-to-G_2_ distance is only 13 mm. For a longer autocorrelation length of 350 nm and hence also longer sample-to-detector distance of 40 mm the incoherent scattering effect already appears to diminish and the increasing SANS based dark-field contrasts according to different sphere sizes become visible in Fig. 2(c).

As also shown in Fig. 2(c), the dark-field contrast images show different contrast depending on the different sizes of polystyrene particles and structures of the cohesive powders. For the diluted polystyrene particle solutions, the dark-field contrast gets stronger as the size of polystyrene particle increases. The top surface of solutions shows saturated contrast due to evaporation because the DFI images are time-averaged. The total measurement time for one result image for each point of the measured autocorrelation length was at about 3 hours and the solutions in the cuvettes evaporated although the cuvettes were sealed by teflon tape. This can also easily be seen from the solution heights in Fig. 2(b,c), where the time gap between the two images is at least about 6 hours. The dark-field contrast of the cohesive powders also shows enhanced contrast for larger powder particles and at longer autocorrelation length, just like for the diluted sphere samples.

### Incoherent scattering contribution and correction

For a quantitative dark-field contrast imaging analysis of the diluted polystyrene particle solution, the incoherent scattering by the aqueous solution has to be taken into account. The H_2_O in aqueous solution has a huge incoherent scattering cross-section which contributes significantly to the reduction of visibility, which obscures the coherent scattering from the structural features of the particles. The incoherent scattering from H_2_O is isotropic and hence reduction of visibility occurred at sample area and background area and resulted in an increased dark-field contrast. The number of incoherently scattered neutrons which are contributing on the detector drops by the inverse-square law according to the sample-to-detector distance. Consequently, the incoherent scattering contribution is dominant at short autocorrelation lengths where the sample is close to G_2_. The total neutron scattering cross-section in Eq. (1) can therefore be separated into a coherent and an incoherent effective neutron scattering cross-section. Hence the correction due to incoherent scattering for diluted polystyrene particle solution can be performed by normalizing the dark-field contrast of the diluted polystyrene particle solutions with the dark-field contrast of a reference solution with the same ratio of H_2_O and D_2_O as the one used for the actual sample. The dark-filed contrast corrected for incoherent scattering finally represents the structural features.

**Figure 3:**
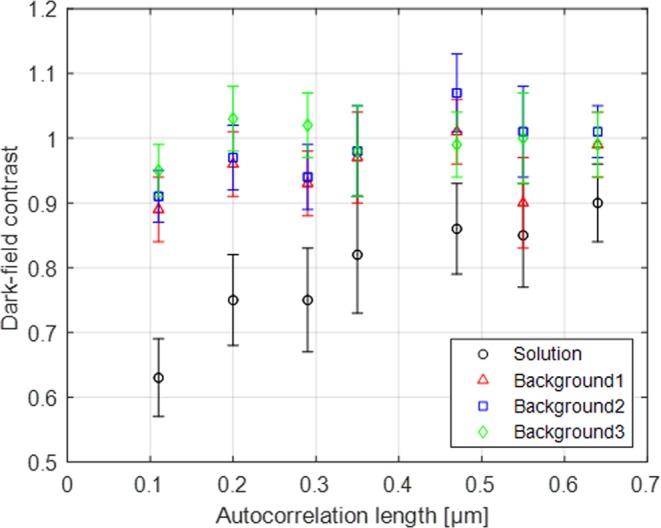
The dark-field contrast along autocorrelation length. Aqueous solution and backgrounds show incoherent scattering from H_2_O in aqueous solution following inverse-square law.

The dark-field contrast of pure aqueous solution and backgrounds along autocorrelation length is shown in Fig. 3. The region of interest (ROI) for the contrast of aqueous solution was selected by 40 × 250 pixels and that for each background region by 20 × 210 pixels. The error bars shown in Fig. 3 are the standard deviations for each ROI. The pure aqueous solution clearly shows dark-field contrast due to the incoherent scattering of H_2_O. The contrast starts from 0.63 at autocorrelation length of 110 nm and decreases along the autocorrelation length following inverse-square law. The background 1 and 2 are two areas between the solutions and background 3 is beside the cohesive powder sample far from solutions (compare Fig. 2). The theoretical dark-field contrast of the background is 1, but the measured contrast of the background is significantly below 1 in particular at shorter autocorrelation lengths probed. This can be related to the incoherent scattering from the aqueous solution. Even background 3, despite at a distance from the aqueous samples, displays at the shortest autocorrelation length a measurable background deviation.

### Quantitative dark-field imaging of diluted polystyrene particle solutions

**Figure 4:**
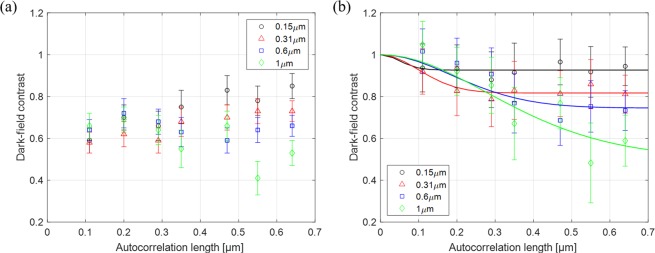
The dark-field contrast along autocorrelation length. (**a**) Diluted polystyrene particle solutions before incoherent scattering correction and (**b**) after incoherent scattering correction. The solid lines represent fit curves from measured data.

The results of dark-field SANS imaging of the diluted polystyrene particle solutions, pure aqueous solution and backgrounds are shown in Fig. 4. The dark-field contrast can be achieved from autocorrelation lengths as short as of 110 nm due to the extended range of symmetric Talbot-Lau neutron grating interferometer by the G_2_ period of 50 μm and the sample-to-G_2_ distance of 13 mm. The region of interest for the contrast of each solution was selected by 40 × 250 pixels and of each background region by 20 × 210 pixels.

The dark-field contrast of the diluted polystyrene particle solutions before incoherent scattering correction is shown in Fig. 4(a). The error bars shown in Fig. 4(a) are the standard deviations for each ROI. The overall dark-field contrast measured versus the set-up autocorrelation length is a result of both the coherent scattering from the diluted polystyrene particles and the incoherent scattering, particularly from H_2_O in the aqueous solution. While based on the isolated sphere model the dark-field signal from coherent scattering starts from full visibility at *ξ* = 0 and saturates at a certain contrast related to Σ, where the autocorrelation length equals to the sphere size^18,27^, the incoherent scattering here obscures the coherent scattering function inducing a contrast as low as 0.6, lower than at coherent scattering saturation, already at the shortest probed autocorrelation length. Only for the larger particles, the coherent scattering is dominant at least in the critical region of the characteristic structure size. However, the analysis of the coherent scattering for structural refinement is not possible without taking into account the incoherent contribution.

Figure 4(b) shows the dark-field contrast curves of the diluted polystyrene particles after incoherent scattering correction. The correction was achieved by simple division of the measured contrast of the polystyrene particle solutions by the measured dark-field contrast of pure aqueous solution. Hence, the error bars shown in Fig. 4(b) are calculated by the error propagation from the standards deviations for each ROI of the aqueous solution and polystyrene particle solutions. The presented model fits, the solid lines in Fig. 4(b), are nonlinear least square fits of the isolated sphere model using the a priori known sphere size as an input parameter. The data is time averaged with respect to the evaporation and thus a changing particle volume fraction is returned as an average in the fitting parameters. Assuming the solution of H_2_O and D_2_O evaporating in same ratio, the neutron scattering cross-section which is extracted by fitting implies an increase of volume fractions of particles from the nominal 6% to 9% for spheres of 0.15 μm and 0.6 μm, and to 12% for 0.31 μm and 1 μm. These are significant changes of 50–100%, which are, however, supported by the corresponding amount of evaporated liquid.

### Quantitative dark-field imaging of cohesive powders

**Figure 5:**
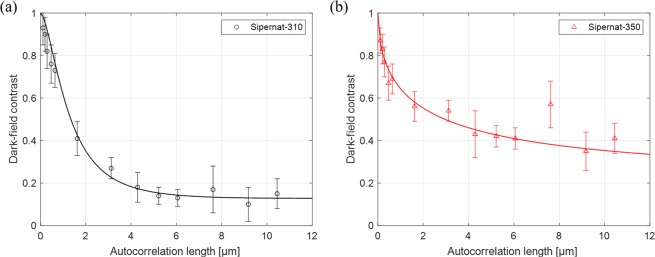
The dark-field contrast of cohesive powders along autocorrelation length. The structure is described by one-dimensional projected real space correlation functions G of random two-phase media. (**a**) The Sipernat-310 with high surface area is presented by a perfectly random distribution of a solid material case and (**b**) the Sipernat-350 with low surface area is presented in simplified case.

The result of dark-field SANS imaging of cohesive powders is shown in Fig. 5. The error bars shown in Fig. 5 are the standard deviations for each ROI. Herein the dark-field contrast can be achieved to the autocorrelation length of 10.45 μm by an extreme sample-to-G_2_ distance of 1187.5 mm. The solid lines in Fig. 5 represent a nonlinear least square fit of a perfectly random distribution of solid material and a simplified case of the random two-phase media model according to the cohesive powders of Sipernat-310 and Sipernnat-350, respectively. According to the information provided by the manufacturer, the cohesive powder Sipernat-310 features a powder particle size of 8.5 μm and has a high surface area of 700 m^2^/g and Sipernnat-350 displays mean particle sizes of 4 μm and has a lower surface area of 55 m^2^/g. This implies that the powders have significantly different structural features. The cohesive powder with 8.5 μm particles features larger particles than Sipernnat-350 with 4 μm particles, but having a higher surface area means that Sipernat-310 has higher dimensionality of fractal structure as well. Therefore, Sipernat-310 is described as a perfectly random distribution (Eq. (8)), as has been shown to be appropriate in previous studies, while Sipernnat-350 is described by a simplified fractal model given in Eq. (9).

The fitting process is conducted with a priori known parameters of packing fraction which is calculated by the initial slope of dark-field contrast along autocorrelation length. The dark-field contrast in an initial range of autocorrelation length can determine the packing fraction of powder. The initial slope is constant, which means the differential of logarithm of dark-field contrast for autocorrelation length can be related to quantity multiplied by the sample thickness, the neutron wavelength, the difference of neutron scattering length density, and the packing fraction. In the calculation of packing fraction, the initial five measured data points were used and the calculated packing fractions were 0.39 × 10^–2^ for Sipernnat-310 and 0.67 × 10^–2^ for Sipernnat-350.

As shown in Fig. 5, the fitting results show reasonable agreement in both cases and underline the different fractal structure and corresponding models required for each case of cohesive powder. The extracted structural parameters of the fractal nature of the cohesive powders from the fitting process are shown in Table 2.

**Table 2:** The structural parameters of fractal nature of the cohesive powders.

Powder	Case	*H* (0 ≤ *H* ≤ 1)	*a* [μm]	*a* (0 < *α* < 2)
Sipernnat-310	Perfectly random distribution of solid material case (Eq. (8))	0.5	1.561	—
Sipernnat-350	Simplified case (Eq. (9))	0.031	7.042	0.536

For Sipernnat-350 the Hurst exponent *H* is 0.031 under 1/2, which represents generally a short-range correlated structure. The measure of size of heterogeneities *a* which is considered as voids in the laminated structure of the powder the in cuvette is 7.042 μm. The structure of phase boundary *α* which represents the dimensional distribution is 0.536 in the range between 0 and 1, which represents open and branched distribution.

The Hurst exponent *H* of the cohesive powder Sipernnat-310 for the perfectly random distribution is fixed to 0.5 but the Hurst exponent Sipernnat-350 being higher than for Sipernnat-310, may indicate long-range correlated structures in the lamination of powder in the cuvette by the size of powder particles. The size of heterogeneities *a* of Sipernnat-310 is 1.561 μm which corresponds well to the findings of different previous studies^29^.

## Conclusions

We have introduced the application of a symmetric Talbot-Lau neutron grating interferometer which provides a significantly extended autocorrelation length range. The symmetric Talbot-Lau grating interferometer allows a quantitative dark-field contrast imaging for the structure in a regime from some ten nanometers to ten micrometers, i.e. three orders magnitude, due to the features of the symmetric set-up, large grating periods and long inter-grating distances. The autocorrelation length range experimentally has been proved from about 100 nm to about 10 μm through the variation of sample-to-G_2_ distance and the highly efficient set-up definitely overcomes the limitation of conventional neutron Talbot-Lau techniques. The quantitative dark-field contrast analysis is demonstrated by reference sample systems of the diluted polystyrene particles in aqueous solutions and two kinds of cohesive powders. Concerning diluted polystyrene particles in aqueous solution, we have demonstrated and discussed the incoherent scattering from aqueous solution dominant at short autocorrelation lengths for correct analysis of the structural feature. Finally, through the dark-field contrast imaging with respect to real space correlation function *G* the structural feature of isolated spheres in a size range below 1 μm has been achieved at autocorrelation length ranges spanning several hundreds of nanometers. In addition, two kinds of cohesive powders have been analyzed by random two-phase media models, and their significant structural parameters and differences of fractal nature have been quantified. In conclusion, the symmetric Talbot-Lau neutron grating interferometer provides access to a significantly extended autocorrelation length range in quantitative dark-field contrast imaging which enables conclusive spatially resolved SANS studies. This novel set-up will allow exploring more accurately and independently structural features of complex heterogeneous samples and thus has great potential value for material science.
